# A comparison of SIEVE, SORT, and START triage training effectiveness between immersive interactive 3D learning materials using virtual reality (VR-SSST) and traditional methods in mass casualty incidents

**DOI:** 10.1186/s12245-025-00850-2

**Published:** 2025-03-13

**Authors:** Kritsada Chumvanichaya, Chaiyaporn Yuksen, Promphet Nuanprom, Kasamon Aramvanitch

**Affiliations:** 1 Division of Emergency Medicine, Department of Emergency Medicine, Faculty of Medicine, Ramathibodi Hospital, Mahidol University, Bangkok, 10400 Thailand; 2 Division of Paramedicine, Department of Emergency Medicine, Faculty of Medicine, Ramathibodi Hospital, Mahidol University, Bangkok, 10400 Thailand

**Keywords:** Virtual reality, Disaster triage, Sieve triage, Sort triage, Start triage, Mass casualty incidents, ARCS model

## Abstract

**Introduction:**

Disaster triage is a crucial competency for paramedics. Traditional training methods, such as lectures and tabletop exercises (TTx), may not provide immersive and high-pressure experience necessary for optimal skill development. Virtual reality (VR) is innovative, allowing trainees to engage in realistic triage simulations in a controlled, interactive environment.

**Objective:**

The study aimed to compare the effectiveness of VR-based triage training and traditional methods by assessing knowledge, learner motivation, and practical skills through pre-and post-tests, the ARCS model, and live simulations.

**Methods:**

This method-oriented, randomized study was conducted over a 2-week intervention among 83 paramedic students and compared traditional lecture-based (*n* = 41) with VR-based (*n* = 42) training for MCI triage education among paramedic students at the Faculty of Medicine, Ramathibodi Hospital, Mahidol University. Both groups attended lectures. Knowledge was assessed through validated pre- and post-tests in four domains: memory, comprehension, application and analysis. Learner motivation was evaluated using the ARCS model (Attention, Relevance, Confidence, Satisfaction), and practical skills were measured during live simulations, assessing time use and a validated accuracy score that included triage steps, proper sequencing, and the correctness of triage judgment.

**Results:**

Both groups demonstrated significant improvements in post-test knowledge scores. The VR group scored higher across all ARCS model dimensions: attention (4.78 vs. 4.17, *p* < 0.001), relevance (4.79 vs. 4.37, *p* < 0.001), confidence (4.74 vs. 4.24, *p* < 0.001), and satisfaction (4.71 vs. 4.34, *p* < 0.001). In the practical triage assessment, the VR group achieved higher accuracy in SORT triage (14.39 vs. 12.09, *p* = 0.001) than the traditional group.

**Conclusion:**

Both training methods effectively improved disaster triage knowledge and skills. However, the VR-based method significantly enhanced learner motivation and SORT triage accuracy, suggesting that VR may be a valuable alternative to traditional TTx in disaster triage training.

**Clinical trial number:**

TCTR20241105003. Registration Site: Thai Clinical Trials Registry. URL: https://www.thaiclinicaltrials.org/show/TCTR20241105003.

**Supplementary Information:**

The online version contains supplementary material available at 10.1186/s12245-025-00850-2.

## Introduction

In mass casualty incidents (MCIs), triage is a critical component of emergency response, as the rapid and accurate prioritization of patients can greatly impact survival outcomes [1]. Paramedics, often the first responders in these situations, must possess exceptional triage skills to classify patients according to injury severity and the urgency of medical care needed [2, 3]. To carry out effective MCIs triage, responders require not only theoretical knowledge but also the practical ability to apply this knowledge under high-stress conditions. Given the increasing frequency and severity of natural disasters, accidents, and man-made crises worldwide, there is an urgent need for training programs that comprehensively prepare paramedics to meet these demanding challenges [4].

Triage systems are essential in managing MCIs, as they classify patients according to injury severity and treatment urgency. For example, the START (Simple Triage and Rapid Treatment) system, widely adopted in the United States, evaluates patients based on mobility, respiratory rate, circulation (including capillary refill or radial pulse), and response to basic commands [5, 6]. This system assigns patients to immediate, delayed, minor, or deceased categories. In contrast, the SIEVE triage system, commonly used in Europe and Australia, emphasizes rapid sorting but omits disability assessment. Following the initial triage, the SORT system adds further criteria, including respiratory rate (RR), systolic blood pressure (SBP), and Glasgow Coma Scale (GCS) scores, to refine intervention prioritization [6].

Paramedic students frequently rely on traditional training approaches for disaster triage, such as lectures and tabletop exercises (TTX), to gain foundational knowledge. However, these methods often fail to replicate the high-stress, complex nature of real-life MCIs. A qualitative study examining medical first responders’ (FRs) experiences revealed fundamental shortcomings in current training programs, including a lack of realistic incident scenarios, authentic casualty simulations, interagency collaboration opportunities, and other critical training elements. Responders stressed the importance of training settings that allow controlled errors and suggested that immersive technologies like virtual reality (VR) could effectively bridge these gaps, enhancing skill acquisition and decision-making under pressure [7].

A study by Way et al. demonstrated strong support for VR, with nearly all participants recommending its use for future disaster training. This feedback underscores VR’s potential to significantly enhance disaster preparedness, addressing the gaps left by conventional methods and fostering critical decision-making skills under pressure [8]. VR is particularly valuable due to their immersive, repeatable nature, which enables skill refinement in a controlled environment. This repeatability is especially beneficial in resource-limited settings where live simulations may be impractical. Moreover, performance assessments within these simulated environments effectively measure training impact, reinforcing VR’s role in advancing medical education for MCIs [6].

High-fidelity VR has emerged as a compelling solution to address challenges related to the scalability and realism of traditional training methods. VR offers realistic, repeatable simulations that allow first responders to practice essential skills within a safe, immersive environment [9]. The study showed that participants in the VR group had greater speed and accuracy compared to traditional methods for training tibia intramedullary nailing in a realistic time-tracked operating room scenario. A comprehensive, safe VR platform enhances surgical skill and confidence. Demonstrating this strategy may assist in narrowing the gap and fostering critical ability to make decisions under pressure [10]. However, while VR can closely simulate real-world scenarios, it may only partially replicate the emotional and physiological stress associated with live simulations. This highlights the importance of using VR as a complementary resource to conventional training, effectively bridging the gap between theoretical knowledge and practical application in real-world MCIs [11, 12].

The Immersive Interactive 3D Learning Materials Utilizing Virtual Reality Technology to Enhance SIEVE, SORT, and START Triage Skills in Mass Casualty Situations (VR-SSST), developed by Chumvanichaya et al. (2024), aims to enhance disaster triage competencies—specifically SIEVE, SORT, and START—through immersive 3D virtual reality environments. VR-SSST was employed to evaluate its feasibility among 30 s-year emergency medical technician students. The research followed a three-phase design: adapting content and scenarios, implementing the tool technically, and conducting pilot testing to gather feedback. The VR-SSST program was validated with a high content validity index (CVI = 1) by EMS specialists in Thailand and demonstrated strong feasibility (M = 4.49, SD = 0.88), confirming its value as a reliable and practical training tool. Proper permissions and references for the use of VR-SSST were obtained, ensuring transparency and adherence to academic standards [13]. This study aims to compare the effectiveness of two educational approaches: traditional lecture-based training supplemented by tabletop exercises and VR-based training in preparing paramedic students for disaster triage. The assessment will focus on knowledge acquisition, learner motivation, and practical skills measured through live simulations.

## Methods

### Study design and setting

This study employed a method-oriented, randomized design to compare traditional lecture-based training with VR-based training for MCI triage education among paramedic students at the Faculty of Medicine, Ramathibodi Hospital, Mahidol University.

In Thailand, paramedicine training is a four-year, university-based bachelor’s degree program. The Bachelor of Paramedicine at Ramathibodi Hospital, Mahidol University, was established in 2015, following a backward curriculum design. The first year emphasizes basic sciences, the second year covers foundational paramedicine care, and the third and fourth years focus on clinical training. The Bachelor of Paramedicine program at Ramathibodi Hospital implements a backward curriculum design, which emphasizes outcome-driven education and integrates disaster triage into the fourth-year curriculum. Students are provided with a 60-minute lecture that introduces foundational disaster triage concepts, in addition to a focused 20-minute session on the SIEVE, SORT, and START methods, as part of the program. This is proceeded by a 40-minute small-group tabletop exercise which fosters a hands-on, collaborative environment and promotes critical thinking and practical application.

Upon completing the program, graduates must pass a national licensing exam administered by the Thai Paramedical Council. They demonstrate competencies in advanced prehospital care, including managing out-of-hospital cardiac arrest (OHCA) and multiple trauma cases. Their procedural skills encompass endotracheal intubation, cardiac defibrillation, and intravenous drug administration in prehospital care.

### Participants

Participants were recruited from the paramedic student cohort at Ramathibodi Hospital, Mahidol University, with informed consent obtained prior to participation. Students with conditions potentially exacerbated by VR, including epilepsy or photosensitive seizures, recent concussion or traumatic brain injury, and severe motion sickness or vestibular disorders, and students who did not participate in the intervention sessions or had prior experience with the simulator were excluded from the study [8]. A total of 83 paramedic students were randomly assigned to either the traditional training group (*n* = 41) or the VR training group (*n* = 42).

### Randomization/ study protocol

Eligible participants received a detailed explanation of the study protocol, and informed consent was obtained prior to enrollment. Random allocation was performed using sequentially numbered, opaque, sealed envelopes (SNOSE), generated by a computer to ensure unbiased assignment. The randomization was conducted in blocks of six, with stratification based on participants’ experience and undergraduate year level. Participants were assigned to either the traditional or VR training group in a 1:1 ratio. While the study did not employ blinding, the structured randomization protocol, supported by Sealed Envelope Ltd. (2022) [14], effectively reduces selection bias, reinforcing the robustness and credibility of the research methodology. There was no blinding for participants, researchers, or data collectors involved in the study.

In the traditional training group, participants received a structured 40-minute MCI triage curriculum comprising the following instructional components:

1. Didactic Lecture: A 20-minute lecture introduced fundamental MCI triage concepts, covering theoretical frameworks and essential principles (Supplement 1).
2. Collaborative TTx: Following the lecture, participants engaged in a 40-minute small-group (group of 4 participants), interactive TTx, allowing them to collaboratively apply triage protocols in a controlled environment (Supplement 2).
3. Post-Test (Supplement 3) and ARCS Motivation Survey (Supplement 4): After completing the instructional components, participants took a 20-minute post-test to assess knowledge retention and completed an ARCS (Attention, Relevance, Confidence, Satisfaction) survey to evaluate their motivational engagement.
4. Standardized Simulation Evaluation, 1 week after learning (Supplement 5).

In the VR-Based Instructional Group, participants engaged in an 40-minute adapted instructional model that integrated VR technology to enhance experiential learning:

5. Didactic Lecture: Similar to the traditional group, participants received a 20-minute introductory lecture on triage principles (Supplement 1).
6. VR Orientation Session: A 10-minute session familiarizing participants with the VR hardware and software, ensuring comfort with the platform prior to the interactive exercise. During the workshop, participants were shown and instructed how to navigate around in the virtual world, interact with the virtual scene, and do physical assessments according to the SIEVE, SORT, and START triage protocols. Before they began the real interactive exercise, the participants had an excellent understanding of the platform and how it worked owing to this demonstration (Supplement 6).
7. Individual VR Simulation Exercise: Each participant completed a 30-minute individual VR-based triage simulation, providing immersive, scenario-based learning to reinforce triage protocol application in a simulated disaster environment (Supplement 7).
8. Post-Test (Supplement 3) and ARCS Motivation Survey (Supplement 4): A 20-minute post-test and ARCS survey evaluated knowledge retention and motivational factors following the VR training session.
9. Standardized Simulation Evaluation, 1 week after learning (Supplement 5).

All participants completed a final evaluation through a standardized simulation designed to objectively assess their triage competency.

#### Standardized patient scenarios

Each participant conducted triage on 10 standardized patients, with their performance independently evaluated by two specialists to ensure scoring reliability. The standardized patients were recruited and thoroughly trained to effectively present different victim conditions. Rehearsals that demonstrated realistic injury presentations—such as limited movement, abnormal breathing patterns, and various levels of consciousness—were part of training. Based on the simulated injuries, standardized patients were also trained to behave appropriately during physical exams and offer participants consistent cues (Supplement 8).

#### Role of facilitator

In both groups, facilitators played a vital role in delivering the didactic lectures and guiding participants through the activities. In the VR group, facilitators provided a demonstration during the 10-minute orientation session to ensure participants understood how to navigate and interact within the virtual environment effectively.

Protocol-Specific Triage Assessments: Three triage protocols were utilized to comprehensively evaluate participants’ triage skills:

#### Sieve triage protocol

Participants applied the Sieve triage protocol to three victims, demonstrating rapid categorization skills based on injury severity.

#### Sort triage protocol

Using the Sort triage protocol, participants triaged another set of three victims, focusing on prioritizing victims according to treatment and transport urgency.

#### START triage protocol

Each participant applied the START triage protocol to four victims, involving a swift assessment and categorization based on injury severity and immediate needs.

The method part includes a pre-test to assess baseline knowledge that was similar to the post-test but reordered to minimise recall bias. Participants completed the pre-test prior any intervention, and the correct answers weren’t disclosed across training to ensure that learning outcomes were attributed to the treatments rather than existing knowledge or feedback. This method provided assessment consistency and study evaluation integrity. A panel of five experts validated the questionnaire, delivering it a Content Validity Index (CVI) of 1, indicating high validity along with research objectives alignment.

### Data gathering and outcome measures

The questionnaire underwent content validation by a panel of five experts, achieving a Content Validity Index (CVI) of 1, indicating excellent validity and alignment with the study objectives. To ensure that the same participants completed the survey, each participant was required to fill out their unique identification number into the form, allowing verification of survey completion. Additionally, assessments with implications for judgment or evaluation were carefully reviewed and subjected to a feedback process to maintain accuracy and reliability.

The study was conducted over a three-month period. The initial two months were dedicated to preparation, recruitment, and participant allocation. The interventions were executed within a single day, during which participants completed a 20-minute pretest, followed by a 40-minute intervention session, and subsequently a 20-minute posttest coupled with the ARCS motivation survey. One week after the intervention, participants underwent a standardized simulation-based skills performance assessment to evaluate and compare outcomes. The data analysis phase, which spanned two weeks, was undertaken immediately following the completion of the assessments. This structured timeline underscores the methodical approach employed throughout the study.

Performance Metrics: The effectiveness of triage training was measured through the following metrics: (Supplement 9).

#### Mean time per victim for each protocol

The average time taken to triage each individual victim within each protocol (Sieve, Sort, and START) was recorded, assessing time efficiency on an individual victim basis.

#### Mean time per protocol

The mean time taken across all victims within each triage protocol was calculated, providing an overall measure of time efficiency for each protocol.

#### Correctness score

A detailed scoring rubric evaluated procedural adherence, sequencing accuracy, and correct triage outcomes, resulting in a composite score to gauge triage accuracy and overall effectiveness.

#### Knowledge and motivation assessments

Knowledge was assessed through a 20-question multiple-choice test covering key triage principles. Knowledge acquisition was measured through pre- and post-tests, evaluating memory, comprehension, application, and analysis skills. Learner motivation was assessed using the ARCS model, which captured engagement, relevance, confidence, and satisfaction through a post-training survey. After the intervention, participants were given a 5-point scale for each topic within ARCS model to evaluate motivation.

#### Live simulation assessment

One week after the training, performance was assessed using a standardized performance matrix on ten simulated patients, divided into three for SIEVE triage, three for SORT triage, and four for START triage. Two independent experts evaluated participants’ performance based on the accuracy of triage decisions and the time taken to complete each task, providing a comprehensive measure of skill retention and practical application in simulated mass casualty incident conditions.

### Definitions

#### Prehospital trauma triage tool

##### Sieve and Sort triage

The Sieve triage system, used in Europe, Australia, and the UK, rapidly prioritizes casualties by evaluating their walking, respiratory rate, capillary refill time, and radial pulse. Ambulatory patients are labeled as “green” (priority3, or delayed), while others are evaluated for vitals and categorized into color-coded categories: red (priority 1, or immediate), yellow (priority 2, or urgent), and black (deceased) [6] (Supplement 10).

Sort triage system: Sort Triage, applied as a secondary triage following Sieve, Sort triage classifies casualties according to severity and likelihood of survival using metrics including the Glasgow Coma Scale, respiratory rate, and systolic blood pressure. Cases with a significant severity are prioritized, while moderate and low-risk cases are either stabilized or delayed [6] (Supplement 11).

##### START triage protocol

START Triage (Simple Triage and Rapid Treatment): START, established in the United States, rapidly assesses patients according to their the ability to walk, respiratory rate, distal pulse or capillary refill time, and ability to follow simple commands. Patients are categorized as red (immediate), yellow (delayed), green (minor), or black (deceased) [6] (Supplement 12).

##### ARCS motivation survey

The ARCS Motivation Model, developed by John Keller, improves learner motivation through four key elements: Attention, Relevance, Confidence, and Satisfaction. Attention involves sustaining interest through varied and stimulating materials, whereas Relevance links content to the objectives and experiences of learners. Confidence is developed through explicit objectives and constructive feedback to enhance learners’ confidence in their success. Satisfaction can be achieved by reinforcing learning achievements with positive reinforcement, rendering the experience gratifying. This systematic method is extensively utilized in educational and training settings to promote enduring motivation and enhanced engagement.

A structured assessment is frequently employed to evaluate the efficacy of the ARCS Motivation Model through evaluating each component—Attention, Relevance, Confidence, and Satisfaction—using validated questionnaires. These questionnaires generally consist Likert-scale items that measure the extent to which each component is addressed in the learning experience.

The ARCS model of motivational design, developed by John M. Keller in 1987, delineates four essential components that enhance learning motivation: Attention, Relevance, Confidence, and Satisfaction [15]. In 2008, Keller expanded on the model, emphasizing strategies to capture students’ interest (Attention), relate learning to their professional context (Relevance), build self-confidence through appropriately challenging materials (Confidence), and ensure learners experience satisfaction with their accomplishments (Satisfaction). The selection of the ARCS Model for motivation and SORT triage accuracy as assessment measures was guided by their well-established effectiveness and theoretical alignment with the study’s objectives. The ARCS Model, a widely recognized framework in educational research, evaluates learner motivation across four key dimensions: Attention, Relevance, Confidence, and Satisfaction [16]. These dimensions are critical to understanding and enhancing learner engagement and are particularly relevant in immersive learning environments like VR-based training. The ARCS Model has been validated through extensive empirical research, demonstrating its reliability and applicability in assessing motivation in similar educational contexts. Its inclusion in this study aligns with the focus on understanding how VR impacts motivation, an essential factor for the success of educational interventions.

Similarly, the choice of SORT triage accuracy as an assessment measure reflects its relevance as a practical and standardized metric in disaster triage training. SORT triage accuracy directly evaluates the application of learned triage protocols, providing a meaningful indicator of the participants’ ability to prioritize patients effectively in high-pressure scenarios. This measure is well-suited for gauging the effectiveness of training interventions in emergency and disaster settings.

A study of Wang LH et al. evaluated the effectiveness of a virtual reality simulation (VRS) triage program in improving nursing students’ learning motivation, attitudes, satisfaction, and experiences, using the ARCS model (Attention, Relevance, Confidence, Satisfaction) to assess motivation. Among 164 third-year nursing students, the VRS group demonstrated significant enhancements in ARCS scores, especially in satisfaction and confidence, compared with the control group adopting traditional case studies. Qualitative findings emphasized issues including practical application, information integration, and skill enhancement, with the primary outcome emphasizing the advancement of professional competency. This research highlights the significance of VRS and the ARCS paradigm in nursing education [17].

### Statistical analysis

#### Sample size Estimation

The sample size for this study was calculated based on findings from Harada et al. [18], which examined the educational effectiveness of self-selected scenarios for triage training using Virtual Reality (VR) in a mass casualty context. This prior study explored the use of VR to enhance the accuracy of the Simple Triage and Rapid Treatment (START) methods among paramedic students, comparing it to traditional lecture-based training approaches.

For the current study, we aimed to detect a moderate effect size, with assumptions of a standard deviation of 1.0, a statistical power of 80%, and a significance level of 5%. Based on these parameters, the initial calculation indicated a required minimum sample size of 32 participants. To account for potential variability and enhance the robustness of the study, a 20% buffer was added, bringing the final required sample size to 39 participants. This adjustment aims to reduce the risk of underpowering the study due to unforeseen participant dropout or data loss, thus supporting the reliability of the findings.

#### Statistical analysis

Statistical analyses were conducted using STATA software version 16.1 (Stata/MP 16.1 for Mac) (StataCorp, College Station, TX, USA). For categorical data, Fisher’s exact test and chi-square tests were applied to assess differences between two independent groups. For dependent proportions, the Exact McNemar’s test was used.

For continuous data, comparisons between two independent means with normal distribution were performed using Student’s t-test with equal variances (for similar SDs) and the Welch’s t-test for unequal variances. For data that did not follow a normal distribution, the ranksum test was applied. Comparisons of dependent means used paired t-tests for normally distributed data and the Wilcoxon signed-rank test for non-normally distributed data. A p-value of less than 0.05 was considered statistically significant. A complete case analysis approach was used for all study variables, with no data imputation.

## Results

**Fig. 1:**
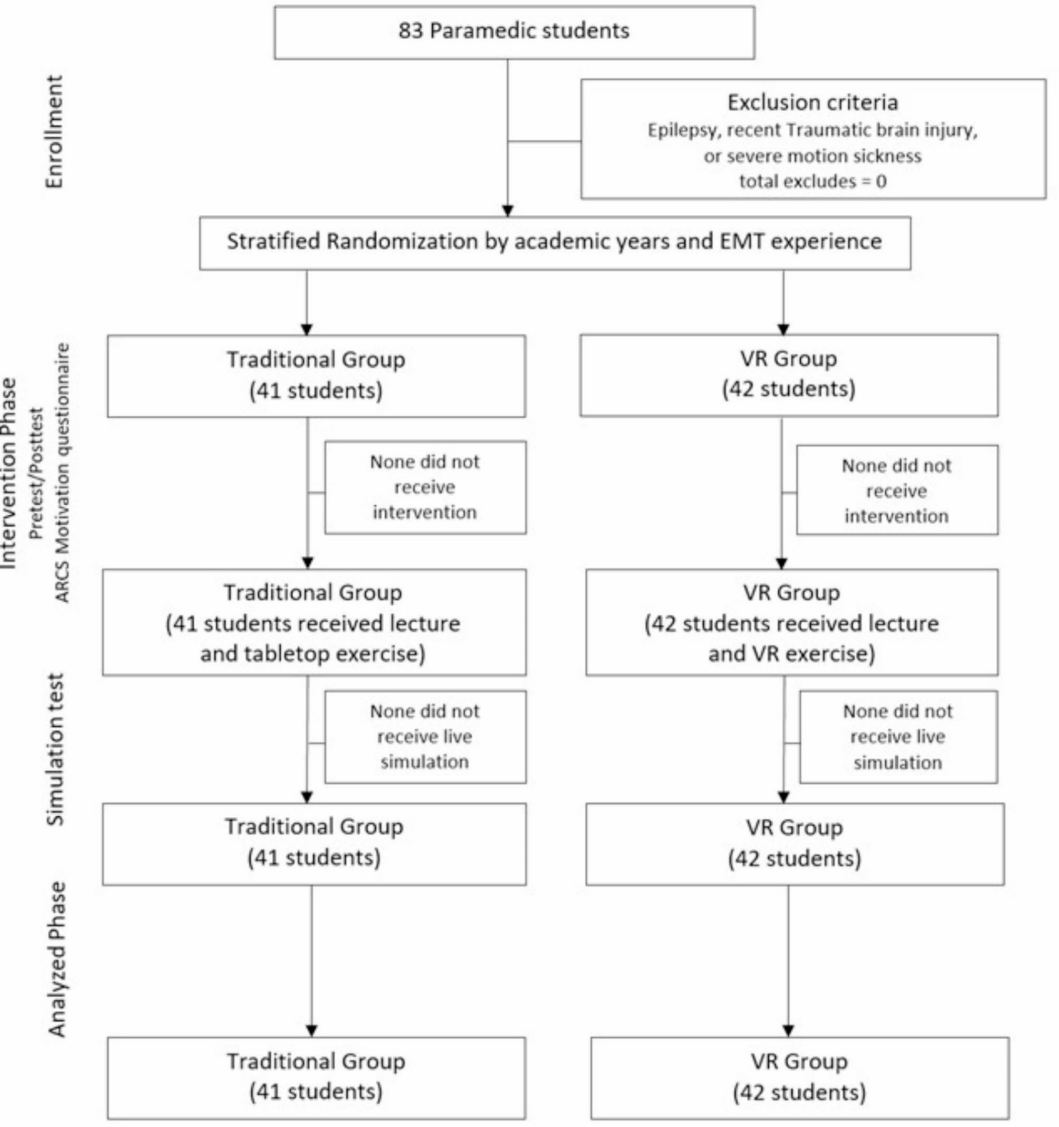
Study flow

A total of 83 paramedic students participated in the study, with 41 assigned to the traditional group and 42 to the VR group. All participants completed both the pre-test and post-test assessments, as well as the final simulation evaluations. There were no dropouts or adverse events reported throughout the study duration. (Fig. 1)

### Baseline characteristics of the study participants

Table 1 presents a comparative analysis of participant characteristics between the traditional group (*n* = 41) and the VR group (*n* = 42), indicating similarity across demographic and experiential variables with no statistically significant differences observed (*p* > 0.05 for all variables).

**Table 1:** Baseline characteristics of the study participants between the traditional group and the VR group

Characteristic	Traditional group (*n* = 41)	VR group (*n* = 42)	*P*-value
Age (mean ± SD)	21.12 ± 1.86	21.43 ± 2.0	0.465
**Academic year; n (%)**			
2	15 (36.59)	15 (35.71)	0.924
3	10 (24.39)	12 (28.57)	
4	16 (39.02)	15 (35.71)	
Prior EMT	6 (14.63)	6 (14.29)	0.604
**Gender**			
Male	13 (31.71)	14 (33.33)	0.530
Female	28 (68.29)	28 (66.67)	
**Experience of triage**			
Sieve triage	20 (48.78)	22 (52.38)	0.457
Sort triage	19 (46.34)	20 (47.62)	0.541
START triage	11 (26.83)	17 (40.48)	0.139
History of VR experience	16 (39.02)	13 (30.95)	0.294

*Abbreviation*: VR: Virtual Reality, SD: Standard Deviation, EMT: Emergency Medical Technicians, START: Simple Triage and Rapid Treatment

The mean age was 21.12 ± 1.86 years in the traditional group and 21.43 ± 2.0 years in the VR group (*p* = 0.465). Physical characteristics were also comparable: the mean height was 166.37 ± cm (SD = 8.731) in the traditional group and 164.86 ± 8.60 cm in the VR group (*p* = 0.430). The mean weight was 63.43 ± 15.71 kg in the traditional group versus 61.48 ± 14.40 kg in the VR group, with no significant difference (*p* = 0.557).

The distribution of academic year was similar between the traditional and VR groups, with most participants in each group representing comparable academic levels, showing no significant differences in distribution (*p* = 0.924). Prior experience as Emergency Medical Technicians (EMTs) was also balanced, with 14.63% in the traditional group and 14.29% in the VR group having prior EMT experience (*p* = 0.604).

Gender distribution was likewise balanced across groups: 31.71% of the traditional group and 33.33% of the VR group were male, while females comprised 68.29% of the traditional group and 66.67% of the VR group, showing no significant gender difference (*p* = 0.530). Prior triage experience was comparable as well, with similar percentages in each group for SIEVE, SORT, and START triage methods. Specifically, 48.78% of the traditional group and 52.38% of the VR group had experience with SIEVE triage (*p* = 0.457). SORT triage experience was held by 46.34% of the traditional group and 47.62% of the VR group (*p* = 0.541), and START triage experience was reported by 40.48% in the VR group compared to 26.83% in the traditional group, though this difference was not statistically significant (*p* = 0.139).

Additionally, previous experience with VR technology was reported by 39.02% of the traditional group and 30.95% of the VR group, without a significant difference (*p* = 0.294). These findings indicate a balanced distribution of demographic and experience-related characteristics between the groups, establishing a well-matched baseline for evaluating training outcomes.

### Pre-test and post-test scores in the traditional and VR groups

Table 2 demonstrates the pre-test and post-test scores for both the traditional and VR groups across four knowledge domains: memory, comprehension, application, and analysis. Both groups showed significant improvements in all domains.

**Table 2:** Pre-test and post-test scores in the traditional and VR groups

Knowledge Test	Traditional group			VR group		
	Pretest	Posttest	*P* value	Pretest	Posttest	*P* value
Knowledge/memory	3.39 ± 1.18	4.20 ± 0.78	< 0.001	3.07 ± 1.37	4.71 ± 0.51	< 0.001
comprehension	3.78 ± 0.99	4.41 ± 0.81	< 0.001	3.67 ± 0.98	4.40 ± 0.89	< 0.001
Application	3.27 ± 1.03	4.32 ± 0.81	< 0.001	3.00 ± 1.23	4.10 ± 0.66	< 0.001
Analysis	2.07 ± 1.19	3.10 ± 1.00	< 0.001	2.50 ± 1.61	3.21 ± 1.00	0.015

*Abbreviation*: VR: Virtual Reality

For memory, the traditional group’s score increased from 3.39 ± 1.18 to 4.20 ± 0.78, while the VR group exhibited a greater improvement from 3.07 ± 1.37 to 4.71 ± 0.51 (*p* < 0.001 for both). In comprehension, the traditional group’s score rose from 3.78 ± 0.99 to 4.41 ± 0.81, and the VR group’s score increased from 3.67 ± 0.98 to 4.40 ± 0.89, with both changes being statistically significant (*p* < 0.001).

In terms of application, the traditional group improved from 3.27 ± 1.03 to 4.32 ± 0.81, and the VR group improved from 3.00 ± 1.23 to 4.10 ± 0.66,both demonstrating statistically significant increases (*p* < 0.001). For analysis, the traditional group improved from 2.07 ± 1.19 to 3.10 ± 1.00, while the VR group improved from 2.50 ± 1.61 to 3.21 ± 1.00 (*p* < 0.001 and *p* = 0.015, respectively).

Overall, both groups improved in all categories, but the VR group showed slightly greater gains, particularly in memory and application.

### Comparison of ARCS motivation model scores

Table 3 presents a comparison of ARCS motivation model scores between the traditional and VR groups across the dimensions of Attention, Relevance, Confidence, and Satisfaction. The VR group outperformed the traditional group in every category.

**Table 3:** Comparison of ARCS motivation model scores

ARCS Motivation model	Traditional group	VR group	*P*-value
Attention	4.17 ± 0.83	4.79 ± 0.42	< 0.001
Relevance	4.37 ± 0.67	4.79 ± 0.42	< 0.001
Confidence	4.24 ± 0.73	4.74 ± 0.50	< 0.001
Satisfaction	4.34 ± 0.66	4.71 ± 0.51	< 0.001

#### Attention

The traditional group scored 4.17 ± 0.83, while the VR group achieved a significantly higher score of 4.79 ± 0.42) (*p* < 0.001).

#### Relevance

The traditional group received a score of 4.37 ± 0.67, compared to the VR group’s score of 4.79 ± 0.42, indicating a significant difference (*p* < 0.001).

#### Confidence

The VR group demonstrated superior confidence scores with a mean of 4.74 ± 0.50, in contrast to the traditional group’s mean of 4.24 ± 0.73 (*p* < 0.001).

#### Satisfaction

The VR group reported an overall satisfaction score of 4.71 ± 0.51, significantly higher than the traditional group’s score of 4.34 ± 0.66 (*p* < 0.001).

Overall, VR training was found to be more engaging, relevant, and satisfying compared to traditional training, with learners reporting higher levels of confidence.

### Accuracy scores and time usage in triage protocols (Table 4)

**Table 4:** Accuracy scores and time usage in triage protocols

Triage type	Outcome	Traditional group	VR group	*P*-value
SIEVE	Accuracy score	8.52 ± 1.72	8.68 ± 1.47	0.652
	Time	34 ± 22.00	34.60 ± 12.96	0.881
SORT	Accuracy score	12.10 ± 3.96	14.39 ± 1.64	0.001
	Time	38.78 ± 24.08	47.52 ± 17.99	0.064
START	Accuracy score	10.88 ± 5.85	13.07 ± 6.00	0.118
	Time	27.76 ± 17.42	33.29 ± 10.91	0.086

START: Simple Triage and Rapid Treatment

The accuracy score for each triage protocol consists of one point for each step in the protocol, one point for proper sequencing, and one point for the correctness of the triage judgment. This scoring structure assesses both technical execution and outcome accuracy, resulting in a total score for each triage step. Additionally, the time taken for each scenario is recorded for a comprehensive assessment.

#### SIEVE protocol

The correctness scores were comparable between the two groups, with the traditional group scoring 8.52 ± 1.72 and the VR group scoring 8.68 ± 1.47 This difference was not statistically significant (*p* = 0.652). Time usage for the SIEVE protocol was also similar, with the traditional group taking an average of 34 ± 22 s and the VR group averaging 34.60 ± 12.96 s, again showing no significant difference (*p* = 0.881).

#### SORT protocol

The VR group significantly outperformed the traditional group in correctness, scoring 14.39 ± 1.64 compared to 12.10 ± 3.96 for the traditional group (*p* = 0.001). However, the VR group took longer to complete the SORT task, with a mean time of 47.52 ± 17.99 s compared to 38.78 ± 24.08 s for the traditional group; this time difference was not statistically significant (*p* = 0.064).

#### START protocol

In terms of correctness, the VR group scored higher, with a mean score of 13.07 ± 6.00 compared to 10.88 ± 5.85 for the traditional group, although this difference was not statistically significant (*p* = 0.118). The VR group also required more time to complete the START triage, taking an average of 33.29 ± 10.91 s compared to 27.76 ± 17.42 s for the traditional group; this time difference was also not statistically significant (*p* = 0.086).

## Discussion

This study aimed to compare the effectiveness of traditional lecture-based training, supplemented by tabletop exercises, with VR-based training in enhancing knowledge, learner motivation, and practical skills in disaster triage among paramedic students. The results from the knowledge assessment (Table 2) indicate that both training methods significantly improved all knowledge domains: memory, comprehension, application, and analysis. These findings align with prior studies in medical education, which demonstrated that VR training also led to significant improvements in knowledge across various areas, such as tracheostomy care, stroke management, and surgical techniques.

The results suggest that VR’s immersive and interactive nature has the potential to revolutionize knowledge acquisition, particularly for tasks requiring rapid recall and practical application. However, as noted in one study, the effectiveness of VR in improving knowledge retention may vary depending on the subject matter [19]. Overall, the findings underscore the promising future of VR in significantly improving knowledge acquisition in disaster triage training for paramedic students.

Both groups showed significant improvements in the memory domain. The larger gains in the VR group suggest that VR’s immersive and interactive features may enhance memory retention by engaging students more fully in realistic disaster scenarios. This is crucial in disaster triage, where quick recall is vital. These findings are consistent with a study by Szczepocka et al., which investigated immersive VR’s impact on cognitive training for older adults, focusing on visual memory and sustained attention. In this randomized controlled trial with 72 participants aged 65–85, the experimental group engaged in VR-based memory and attention tasks, while the control group experienced non-interactive VR. The study found significant improvements in the experimental group’s visual memory and sustained attention, especially in working memory tasks, underscoring VR’s potential to enhance cognitive skills in diverse populations [20].

Both groups demonstrated significant progress in the comprehension domain. Although the gains were comparable, VR’s immersive experience may offer an advantage by allowing learners to apply theoretical knowledge in realistic scenarios, reinforcing their understanding in a lifelike context. In the application domain, the traditional group’s scores rose from 3.27 to 4.32, while the VR group’s scores increased from 3.0 to 4.10, which is statistically significant. VR’s unique ability to replicate high-pressure, real-life situations likely significantly enhanced practical application skills, effectively bridging the gap between classroom instruction and real-world disaster response. In the analysis domain, scores also improved, with the traditional group moving from 2.07 to 3.10 and the VR group from 2.50 to 3.21, which is statistically significant. Although the gains in analytical skills were less pronounced than in other areas, VR’s interactive elements facilitated slightly better critical thinking and decision-making improvements, especially under simulated pressure. These findings highlight VR’s value in fostering deeper cognitive engagement and practical application, which is essential for effective MCI triage.

These findings align with previous literature on VR’s educational impact. Behmadi et al. (2020) found that while there were no statistically significant knowledge differences between VR and lecture-based methods for teaching START triage, VR offered advantages in knowledge retention and application, suggesting that VR’s immersive qualities may support longer-term learning outcomes [21]. Zhao et al. (2021) observed that students trained in VR environments outperformed traditional methods, highlighting VR’s effectiveness in enhancing memory and practical skills through realistic, scenario-based learning. These reinforce VR’s capacity to support meaningful, lasting learning experiences, especially valuable in high-stakes fields like MCI triage [22].

In our assessment of learner motivation, the VR group consistently outperformed the traditional group across all dimensions of the ARCS model—attention, relevance, confidence, and satisfaction. This outcome is supported by a systematic review by Brown et al. (2023), which found that virtual and augmented reality in disaster medicine training effectively elevated participants’ confidence and satisfaction. VR’s practical, immersive component helps build learner confidence, providing students with a more engaging and enjoyable educational experience than traditional methods [23].

In our study, significant differences in SORT triage accuracy were identified between the VR and traditional training groups, with the VR group showing superior performance. This finding indicates that VR’s immersive, interactive environment better prepares students for complex decision-making in high-pressure settings like MCIs. VR’s ability to recreate realistic disaster conditions likely contributed to these gains, allowing students to practice and refine their skills in controlled yet lifelike scenarios. Similarly, Harada et al. (2024) reported that VR enhanced practical skills in the more straightforward START triage task, underscoring VR’s general utility for practical training. While Harada et al. highlighted VR’s effectiveness for more straightforward tasks, our study demonstrates VR’s added advantage in more complex tasks such as SORT triage. This suggests that VR-based training may be particularly beneficial as task complexity increases, with its effectiveness scaling to meet the demands of higher-level triage processes [18].

Furthermore, no significant differences were observed between the VR and traditional groups regarding accuracy for simpler triage processes, such as START and SIEVE. This indicates that VR and traditional training methods are equally effective in teaching foundational triage skills. Although the VR group took slightly more time to complete these tasks, the additional time—less than one minute—remained within the recommended 30–60 s for disaster triage, especially in START triage. This suggests that either method is appropriate for real-world disaster scenarios where timely decision-making is essential [6]. Paramedic students successfully applied SORT triage principles after VR training by prioritizing patients based on the SORT protocol during a simulated multi-casualty event. The VR training provided realistic physical assessments, such as measuring respiratory rate, checking systolic blood pressure (SBP) displayed on a monitor after taking blood pressure, and evaluating the Glasgow Coma Scale (GCS). This practical experience enhanced their decision-making skills and understanding of the protocol, improving their accuracy in applying SORT triage effectively. VR participants presumably spent more time evaluating their assessments against the actual settings they were trained on. VR may have developed more comprehensive examinations and careful triage due to its immersion. With less experience performing actual physical examinations, the traditional group made quicker decisions but made more errors.

While time efficiency is crucial in disaster settings, accuracy is equally paramount, particularly in high-stakes environments like MCIs. The VR group’s higher accuracy in SORT triage not only shows VR’s effectiveness in complex tasks but also underscores its potential to significantly enhance decision-making. This trade-off between time and accuracy may be warranted in situations where the precision of triage decisions is more important than speed, supporting the use of VR to not just improve but transform competencies in complex emergency scenarios.

## Limitation

This study underscores the advantages of VR-based training but is subject to limitations. First, conducting the study within a single institution limits the generalizability of our findings to other educational contexts. Additionally, the VR group’s slightly longer response times during simulations suggest that VR’s immersive nature could impact decision-making speed—an essential factor in real-world, time-sensitive situations. Future studies should examine whether extended or adaptive VR training can enhance speed and efficiency without sacrificing decision-making quality.

To confirm these results, further studies across diverse educational settings and investigations into the long-term retention of knowledge and skills acquired through VR training are essential. Understanding the durability of VR-based training effects will help assess its value beyond the immediate training period. Additionally, evaluating VR’s cost-effectiveness and scalability could determine its practicality for broader adoption in EMS programs.

The study lacked blinding for participants, researchers, and data collectors, which may have introduced bias in participant behavior and assessments. Future studies should consider incorporating blinding to enhance reliability.

Additional study is required to evaluate the cost-effectiveness of VR-based training relative to traditional techniques, highlighting its scalability, practicality, and capacity to augment or supplant existing approaches in various contexts. Furthermore, the enduring retention of information and abilities acquired by VR training need investigation to evaluate its lasting effects over time. Moreover, subsequent research should explore the adaptation of VR training for collaborative, multi-user settings to support team-based learning and enhance in disaster training situations. Addressing these challenges could promote the comprehensive integration of VR into educational programs and its adoption in complicated training settings.

MCIs necessitate cooperation, communication, and interagency coordination, which single-user VR cannot imitate. Additional research into multi-user VR environments might fill these gaps by including team-based interactions and decision-making, making disaster training more feasible.

Finally, the potential of VR for collaborative, multi-user environments is an inspiring prospect for the future of EMS education. Expanding VR to support such environments could significantly improve training for disaster scenarios such as prehospital trauma care, including training for obstetric emergencies, and basic or advanced life support, VR can provide realistic, risk-free simulations for procedures like CPR and airway management. Traditional single-user VR may not fully capture the collaborative demands of mass casualty incidents (MCIs). However, a study adapting single-player VR to multi-user formats found that collaborative environments improved learner self-efficacy, satisfaction, and confidence. This suggests that developing VR for collaborative training could enhance realism and effectiveness, making it better suited for EMS professionals facing real-world MCIs.

## Conclusion

VR and traditional training methods effectively teach foundational triage skills, especially for simpler protocols like START and SIEVE. However, VR offers distinct advantages in complex tasks such as SORT triage, where decision-making demands are higher. It notably enhances learner motivation and engagement. These findings highlight VR-based training as a valuable addition to disaster triage education, particularly for scenarios requiring advanced decision-making and specialized skill development.

The accuracy and immersive learning environment provided by VR make it a promising alternative to traditional tabletop exercises. Its ability to recreate high-pressure, realistic scenarios positions VR as a powerful tool for future integration into emergency medical education and disaster preparedness programs. By enabling students to practice and refine critical skills in a controlled, lifelike setting, VR-based training has the potential to significantly enhance preparedness for real-world disaster scenarios.

## Electronic supplementary material

Below is the link to the electronic supplementary material.

**Supplementary Material 1: Supplement 1:** A 20-minute didactic lecture was delivered to introduce foundational concepts of MCI triage

**Supplementary Material 2: Supplement 2:** 40-minute small-group Tabletop exercise

**Supplementary Material 3: Supplement 3:** 20-minute post-test to assess knowledge retention

**Supplementary Material 4: Supplement 4:** ARCS (Attention, Relevance, Confidence, Satisfaction) Motivation Survey

**Supplementary Material 5: Supplement 5:** Standardized Simulation Evaluation, 1 week after learning

**Supplementary Material 6: Supplement 6:** A 10-minute Virtual reality Orientation Session

**Supplementary Material 7: Supplement 7:** Individual VR Simulation Exercise

**Supplementary Material 8: Supplement 8:** Standardized Patient Scenarios: Each participant conducted triage on 10 standardized patients, with their performance independently evaluated by two specialists to ensure scoring reliability

**Supplementary Material 9: Supplement 9:** Performance Metrics: The effectiveness of triage training was measured through the following metrics

**Supplementary Material 10: Supplement 10:** Sieve triage protocol

**Supplementary Material 11: Supplement 11:** Sort triage protocol: GCS evaluation

**Supplementary Material 12: Supplement 12:** Start triage protocol

## Data Availability

The datasets used and/or analyzed during the current study are available from the corresponding author on reasonable request.

## Declarations

### Using artificial inteligence chatbots

During the preparation of this work the author(s) used ChatGPT4.0 in order to check and correct grammatical error during the manuscript writing process. After using this tool/service, the author(s) reviewed and edited the content as needed and take(s) full responsibility for the content of the publication.
