# Supplementary figures and images for: A comparison of SIEVE, SORT, and START triage training effectiveness between immersive interactive 3D learning materials using virtual reality (VR-SSST) and traditional methods in mass casualty incidents
Source: Int J Emerg Med. 2025 Mar 13;18:55. doi: 10.1186/s12245-025-00850-2 (PMC11905642; doi:10.1186/s12245-025-00850-2)

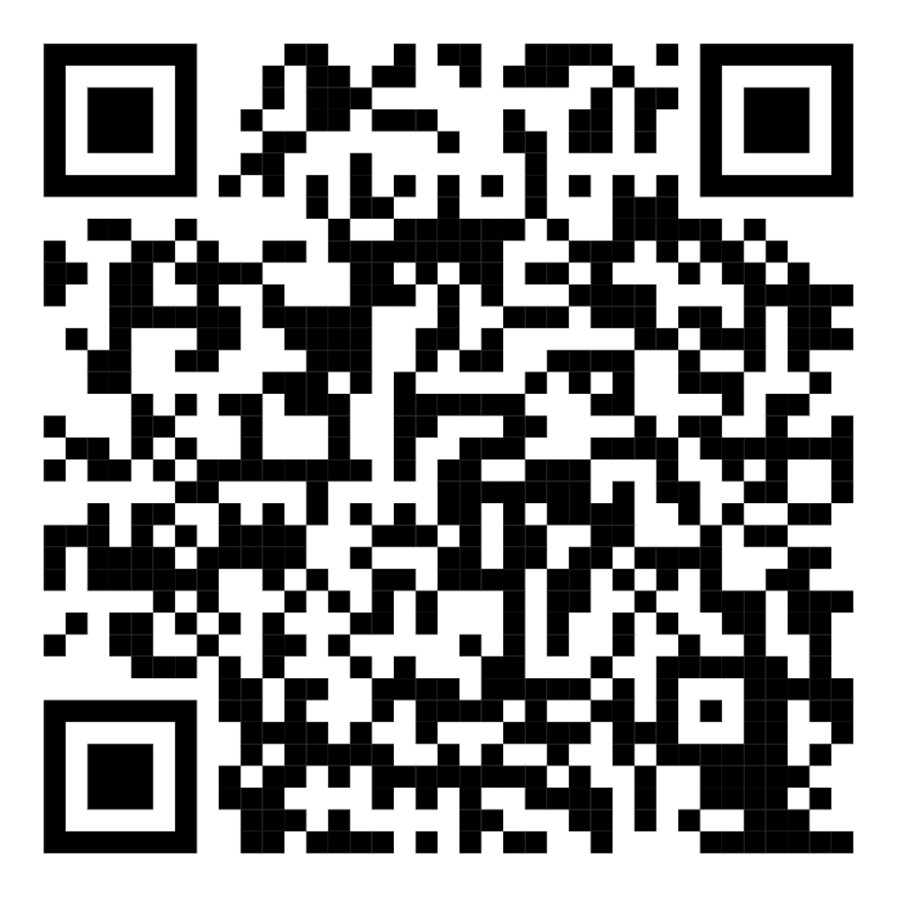

Supplement: Supplementary file 1 — Supplementary Material 1: Supplement 1: A 20-minute didactic lecture was delivered to introduce foundational concepts of MCI triage [file 12245_2025_850_MOESM1_ESM.jpg]

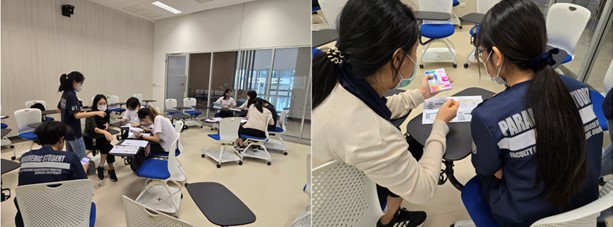

Supplement: Supplementary file 2 — Supplementary Material 2: Supplement 2: 40-minute small-group Tabletop exercise [file 12245_2025_850_MOESM2_ESM.jpg]

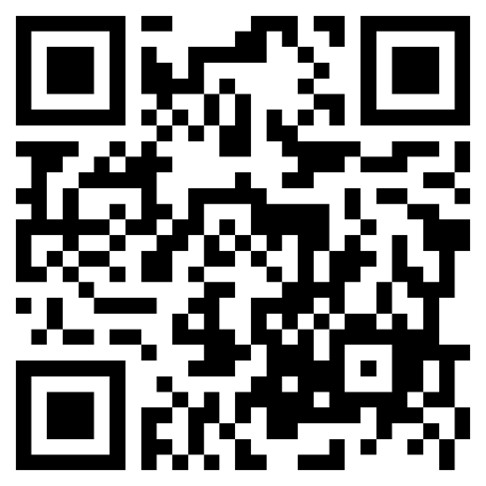

Supplement: Supplementary file 3 — Supplementary Material 3: Supplement 3: 20-minute post-test to assess knowledge retention [file 12245_2025_850_MOESM3_ESM.jpg]

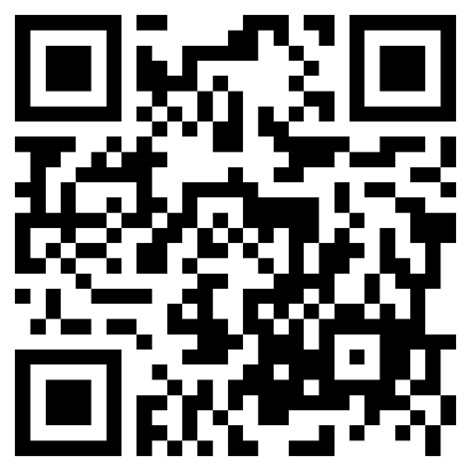

Supplement: Supplementary file 4 — Supplementary Material 4: Supplement 4: ARCS (Attention, Relevance, Confidence, Satisfaction) Motivation Survey [file 12245_2025_850_MOESM4_ESM.jpg]

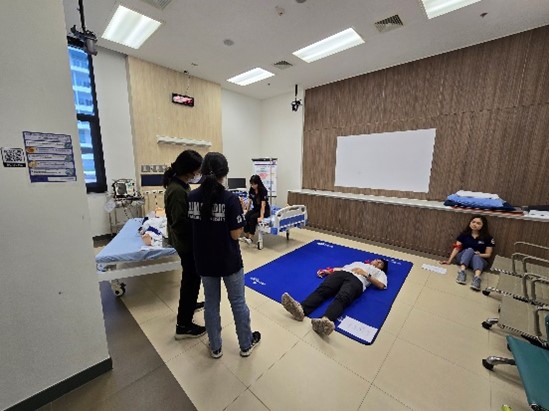

Supplement: Supplementary file 5 — Supplementary Material 5: Supplement 5: Standardized Simulation Evaluation, 1 week after learning [file 12245_2025_850_MOESM5_ESM.jpg]

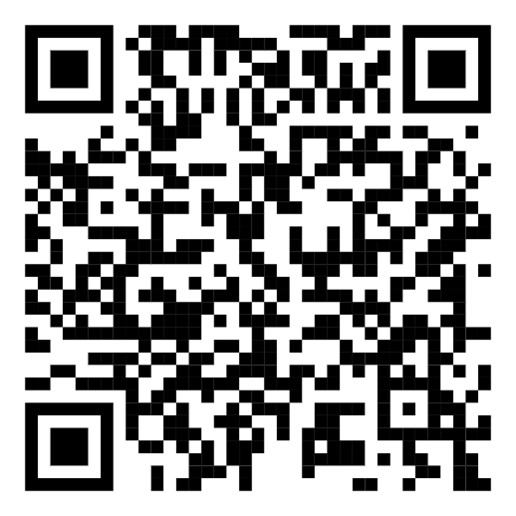

Supplement: Supplementary file 6 — Supplementary Material 6: Supplement 6: A 10-minute Virtual reality Orientation Session [file 12245_2025_850_MOESM6_ESM.jpg]

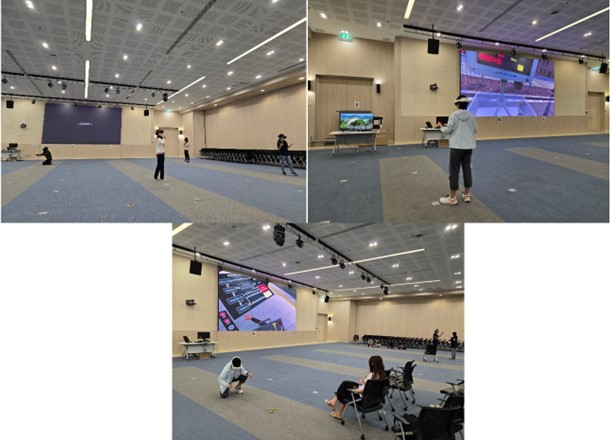

Supplement: Supplementary file 7 — Supplementary Material 7: Supplement 7: Individual VR Simulation Exercise [file 12245_2025_850_MOESM7_ESM.jpg]

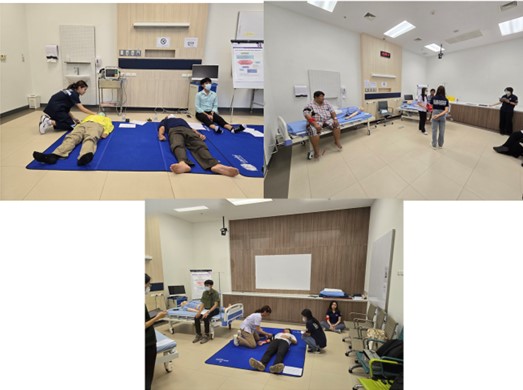

Supplement: Supplementary file 8 — Supplementary Material 8: Supplement 8: Standardized Patient Scenarios: Each participant conducted triage on 10 standardized patients, with their performance independently evaluated by two specialists to ensure scoring reliability [file 12245_2025_850_MOESM8_ESM.jpg]

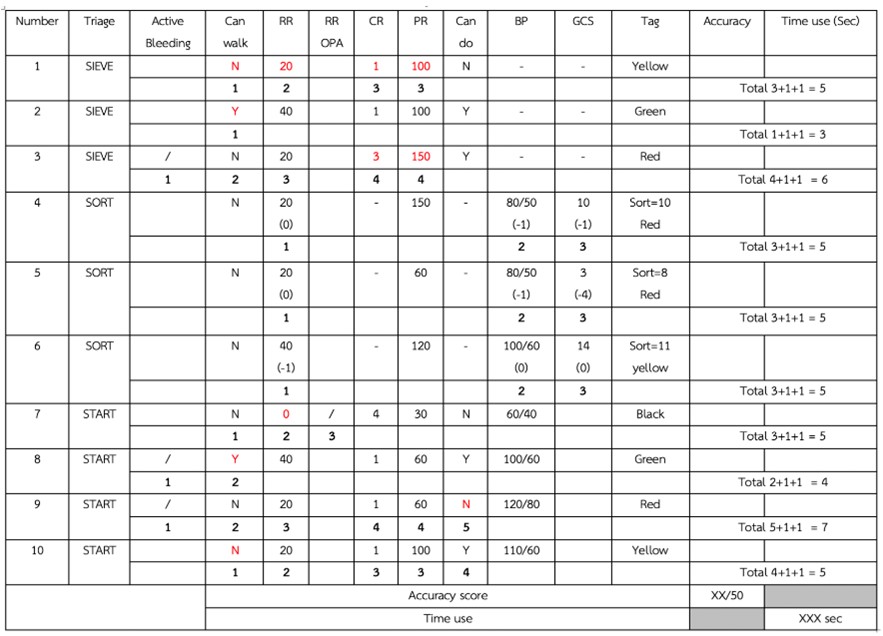

Supplement: Supplementary file 9 — Supplementary Material 9: Supplement 9: Performance Metrics: The effectiveness of triage training was measured through the following metrics [file 12245_2025_850_MOESM9_ESM.jpg]

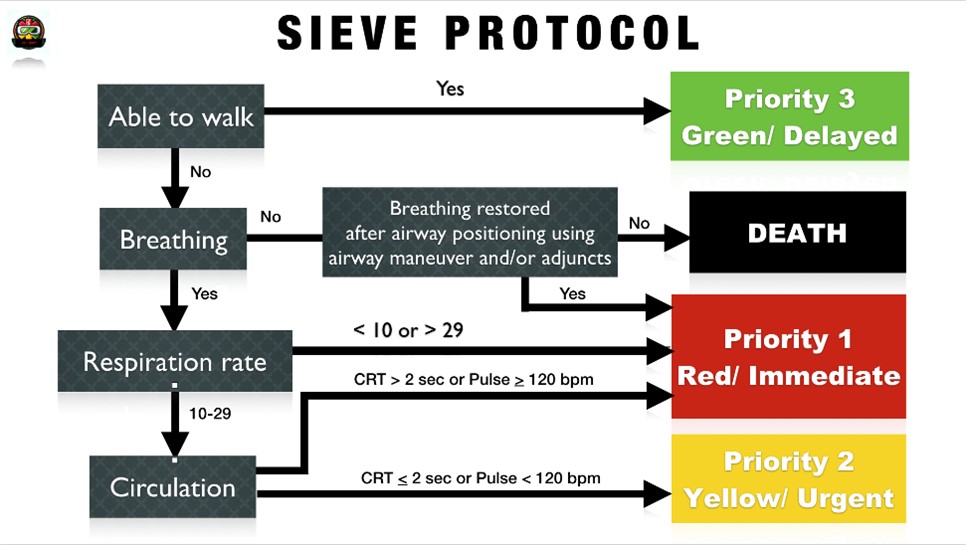

Supplement: Supplementary file 10 — Supplementary Material 10: Supplement 10: Sieve triage protocol [file 12245_2025_850_MOESM10_ESM.jpg]

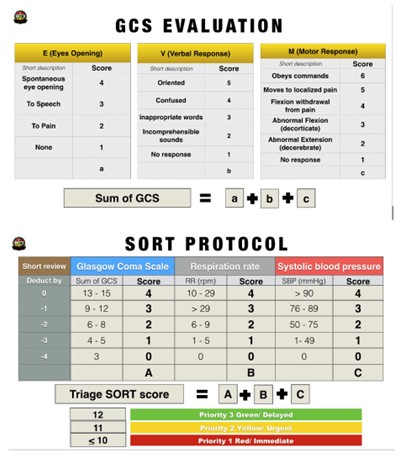

Supplement: Supplementary file 11 — Supplementary Material 11: Supplement 11: Sort triage protocol: GCS evaluation [file 12245_2025_850_MOESM11_ESM.jpg]

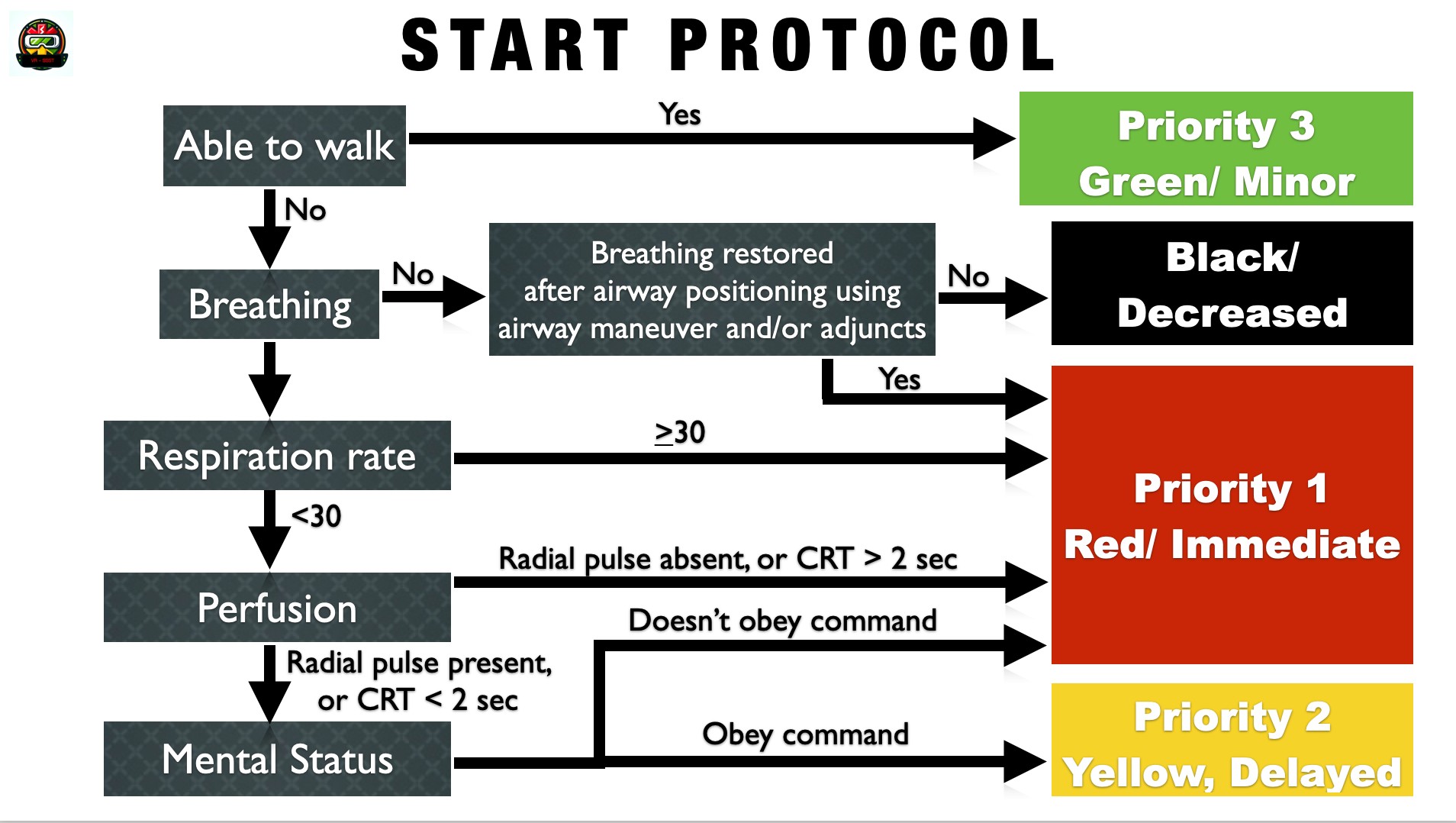

Supplement: Supplementary file 12 — Supplementary Material 12: Supplement 12: Start triage protocol [file 12245_2025_850_MOESM12_ESM.jpg]
